# The examination of the nephroprotective effect of montelukast sodium and N-acetylcysteine ın renal ıschemia with dimercaptosuccinic acid imaging in a placebo-controlled rat model^1^

**DOI:** 10.1590/s0102-865020200090000005

**Published:** 2020-10-16

**Authors:** Arif Aydin, Mehmet Melih Sunay, Tolga Karakan, Serkan Özcan, Ahmet Metin Hasçiçek, İbrahim Yardimci, Hatice Surer, Meliha Korkmaz, Sema Hücümenoğlu, Emre Huri

**Affiliations:** I MD, NEÜ Meram Medicine Faculty Department of Urology, Konya, Turkey. Design of the study, acquisition of data, manuscript writing.; II MD, Professor, Karabük University Medicine Faculty Department of Urology, Karabük, Turkey. Manuscript preparation.; III MD, Associate Professor, Ankara Training and Research Hospital, Clinic of Urology, Ankara, Turkey. Design of the study.; IV MD, İzmir Katip Çelebi University Department of Urology, İzmir, Turkey. Analysis of data.; V MD, Ankara Training and Research Hospital, Clinic of Urology, Ankara, Turkey. Acquisition of data.; VI MD, Adana Çukurova State Hospital, Clinic of Urology, Adana, Turkey. Scientific and intellectual content of the study, acquisition of data.; VII MD, Ankara Training and Research Hospital, Department of Biochemistry, Ankara, Turkey. Analysis of data.; VIII MD, Professor, Ankara Training and Research Hospital, Department of Pathology, Ankara, Turkey. Analysis of data.; IX PhD, Associate Professor, Hacettepe University, Department of Urology, Ankara, Turkey. Manuscript writing.

**Keywords:** Acetylcysteine, Oxidative Stress, Reperfusion Injury, Kidney, Rats

## Abstract

**Purpose:**

To determine the nephroprotective effect of NAC and Montelukast Sodium administration against the development of renal damage associated with long warm renal ischemia.

**Methods:**

Twenty-seven rats were randomly divided into 3 study groups, which received NAC, montelukast and placebo, and 3 rats were included in the sham-treated control group. Medications were given 3 days before the procedure. DMSA renal scintigraphy was performed before and after surgery. The right renal pedicle was occluded for 45 min to induce ischemia and then subjected to reperfusion for 6 h (I/R groups).

**Results:**

On pathological examination, the mean pathological scores of the montelukast and NAC groups were significantly lower than those of the placebo group. (p <0.05). In biochemical examination, significant differences were found in all parameter levels between the placebo group and the montelukast and NAC groups. (p <0.05) When postoperative DMSA renal scintigraphy measurements and renal function levels were compared, significant differences were found between the montelukast and NAC groups and the placebo and sham groups.

**Conclusion:**

The administration of NAC and montelukast sodium was seen to have a nephroprotective effect against the development of renal damage associated with warm renal ischemia.

## Introduction

Causing acute renal damage, renal ischemia can origin morbidity and mortality. Tubular necrosis is the most common cause of renal damage^1^. Decreasing and/or temporary stopping of kidney blood flow may occur due to many factors such as renal transplantation, cardiac bypass surgeries, trauma, partial nephrectomy and sepsis^2^. Although reperfusion, which is defined as the returning of blood flow to the ischemic tissue as it regains normal functions, paradoxical damage may occur due to the free oxygen radicals and other inflammatory mediators forming during reperfusion^3^. After Ischemia Reperfusion (I/R), endoplasmic reticulum damage, autophagosome formation and cell death occurs^4^.

In urology practice, protection against I/R damage and preservation of present nephron capacity is important in partial nephrectomy applied commonly in the treatment of small RCCs and renal transplantation^5^. Many treatment options were presented in literature to minimize nephron damage to occur due to the renal ischemia/reperfusion occurring in urology practice. But none could enter practical application.

Biochemical, histological and scintigraphic evaluation of the nephroprotective effect of montelukast sodium and N-acetylcysteine following one-sided ischemia was planned in this study.

## Methods

All procedures performed in studies involving animals were in accordance with the ethical standards of the institution or practice at which the studies were conducted. Approval for the study was granted by the local ethics committee. The study was performed in the Experimental Animals Laboratory of Ankara Training and Research Hospital (No: 2013/0014).

Thirty male Wistar albino rats (200-250 gr) were located in a room with an air conditioner and at a constant temperature of 22 ± 2°C and a relative humidity of 65–70%, a cycle of 12 hours of light and dark was provided. Approval of Ankara Training and Research Hospital Animal Care and Use Committee was taken for all experimental protocols. Anesthesia was applied in all surgical operations.

### Surgery and experimental protocol

NAC, Montelukast and placebo were the three random groups formed by rats. There were nine rats in each group. Three rats were separated as sham-treated control group. Pre-operative medical treatments were started three days before. As medication, 200 mg/kg NAC in NAC group and 10 mg/kg Montelukast in montelukast group were added to the drinking water per day. Medication was continued until the rats were sacrificed. Although renal ischemia was formed, the placebo group was not given any medication. Sham group did not have renal ischemia and treatment.

**Figure 1:**
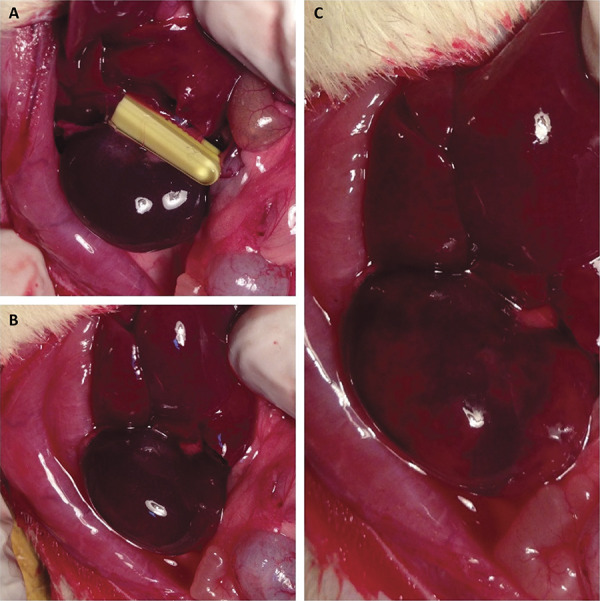
- A. Renal ischemia with atraumatic microvascular clamp on the renal pedicle. B. Postischemic kidney image. C. Ischemia-reperfusion kidney image.

All rats had DMSA renal scintigraphy before the surgical operation and after 6-hour reperfusion. A combination of 40 mg/kg ketamine HCl (Parke-Davis) and 10 mg/kg xylazine HCl (Bayer, Leverkusen, Germany) was applied intramuscularly in the rats. After shaving the incision area, a sterile environment was provided by cleaning with povidone iodine. Right kidney was reached through laparotomy via midline incision. Right renal pedicle was isolated and renal ischemia was formed through right renal pedicle occlusion for 45 minutes. Renal reperfusion was provided for six hours after ischemia (I/R groups) (Fig. 1). DMSA renal scintigraphy was repeated after reperfusion. Left nephrectomy was applied 24 hours before sacrification in all groups except Sham group. Right nephrectomy was applied and blood of the rats was taken before sacrification. Right kidneys were histopathologically and biochemically evaluated. Including MDA and SH levels which are lipid peroxidation indicators and oxidative stress markers in the tissue, MDA and SH levels were checked. Blood urea and creatinine were also measured.

### Biochemical analysis

Animals were sacrificed 24 hours after nephrectomy. Blood urea and creatinine were measured by taking intracardiac blood sample to evaluate renal functions. Some of the renal tissues taken were kept at -80^o^C for biochemical evaluation. Histopathological evaluation was performed by locating remaining renal tissue in 10% formaldehyde. In the renal tissues kept, as the demonstrator of indirect neutrophil infiltration, Malondialdehyde (MDA), an end product of lipid peroxidation, glutathione (GSH), a key antioxidant, and tissue-associated myeloperoxidase (MPO) activities and total nitrite levels as free oxygen radical demonstrator were measured. Automatically, serum urea and creatinine levels were determined spectrophotometrically (Olympus AU 2700).

### Histopathological analysis

10% formalin solution was used for fixating the kidney tissues of sacrificed rats. Renal cortex and renal pelvis macroscopic sections were taken after standard preparation; a paraffin block was formed and cross-sections with a thickness of 5 mm were taken with microtome. Preparations were routinely stained with H&E and the groups were evaluated by the same blinded pathologist.

**Figure 2:**
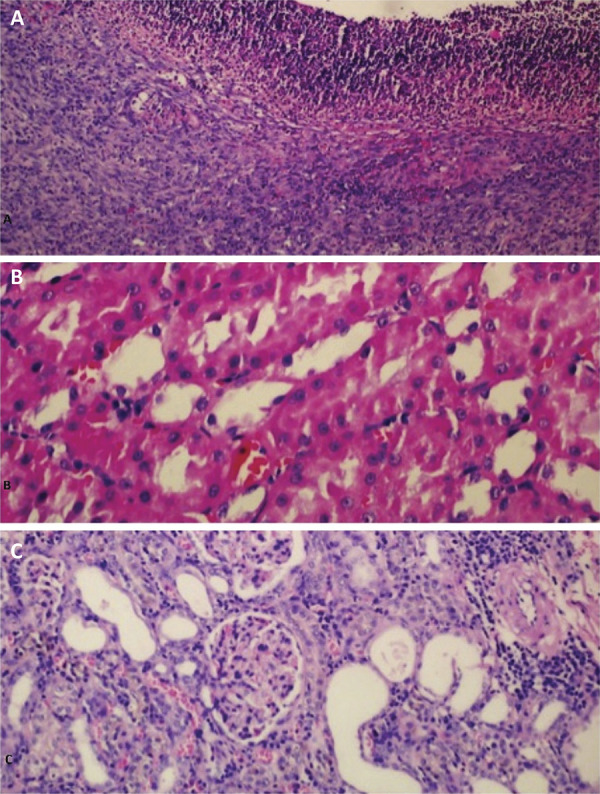
A. Ulceration of the urothelium, extensive fibrosis of subepithelial tissue and moderate chronic inflammation (HE x200). B. Areas of unaffected tubules displaying normal nuclei with open chromatin, retained brush borders and cytoplasmic integrity (HE x400). C. Mild chronic interstitial inflammation (HE x200).

For histological evaluation, previously defined criteria were used with scoring of 0= normal histology, 1 point =swelling in the tubular cells, bruised edge loss and up to a third nuclear condensation, 2 points=in addition to the 1-point changes, tubular changes of one-third to two-thirds, and 3 points =tubular changes of more than two-thirds. Based on these criteria, all kidneys were evaluated at 100 sites and were scored up to a maximum of 300 points (Fig. 2).

### DMSA renal scintigraphy

**Figure 3:**
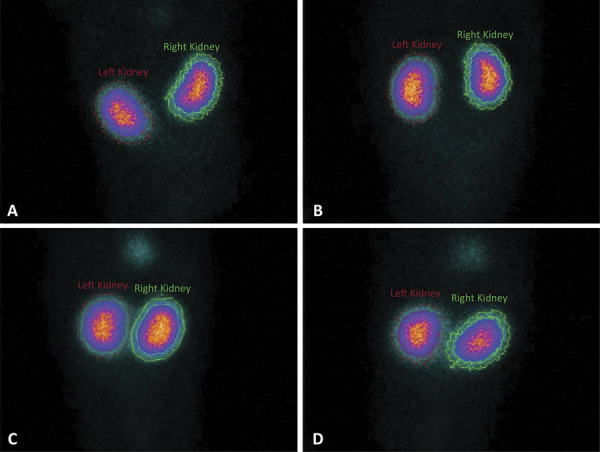
Preoperative (A) and postoperative (B) DMSA scintigraphy in posterior image of a rat in the control group (uptake value of the right kidney; preoperative = 49.49%, postoperative = 45.32%), Preoperative (C) and postoperative (D) DMSA scintigraphy in posterior image of a rat in the Montelukast group (uptake value of the right kidney; preoperative = 50.43%, postoperative = 48.97%).

Using e.CAM gamma camera system (Siemens, Erlangen, Germany), the rats had DMSA renal scintigraphy pre-operatively and 6 h after reperfusion. All rats were anesthesized through the intramuscular injection of ketamine and 2% xylazine. Then, 99m-Tc-DMSA was intravenously administered on a tail vein at a dose of 37 MBq (1 mCi). Images were taken after two hours. At a 140 keV energy peak in a 20% window and at 2-minute intervals with anterior and posterior static, images were recorded using a pinhole collimator. At x2.67 magnification in a 256 matrix, planar static images were taken. Regions of interest (ROI) were drawn on both kidneys and were used for the evaluation of front and back static images. Using ROI-obtained numerical values in the anterior and posterior positions, separate calculations were performed for the geometric means of the contributions to total renal function. Thus, percentage functional contribution of each kidney was determined (Fig. 3).

### Statistical analysis

Data obtained in the study were analysed statistically using SPSS for Windows, version 10.0 software. Kruskal-Wallis and Mann-Whitney U tests were applied for the comparisons. In statistical evaluations, p <0.05 was considered as the statistically significant value.

## Results

None of the animals died throughout the experiment. There were no complications after surgery. The groups were scored microscopically in histopathological property, biochemically for oxidative stress markers and Nuclear Medicine for DMSA renal scan.

### Microscopic evaluation results

No pathological changes were observed in Sham group. Mean pathological score was calculated as 41.11±6.91 in montelukast group and there was no substantial difference from NAC and Sham groups (p> 0.05). But the pathological scoring was significantly lower than the placebo group (p < 0.05). Mean pathological score was calculated as 31.11±8.92 in NAC group, and while there was no significant difference from Montelukast and Sham groups (p> 0.05), it was importantly lower than the placebo group (p <0.05). Placebo group’s pathological score was detected meaningfully higher than Sham and placebo groups (p <0.05) (Table 1).

**Table 1:** Postoperative DMSA renal scintigraphy and pathology assessment.

	Patology (Mean[SD])	Dmsa Postop(%) (Mean[SD])
Montelukast Group	(41.11[6.91])	(45.27[.73])
NAC Group	(31.11[8.92])	(44.13[.605])
Plasebo Group	(97.22[13.07])	(39.04[.490])
Sham Group	(26.67[4.41])	(50.20[.554])
P values*		
Montelukast vs plasebo group	.000	.000
NAC vs Plasebo Group	.000	.000
Montelukast vs sham treated	.114	.013
NAC vs sham treated	.852	.013

*Right kidney function only.

For patology and DMSA overall group comparisons Mann-Whitney U and Kruskal wallis test *P<.05.*

### Biochemical evaluation results

Mean antioxidant SH amount was detected as 45.09±6.73 in montelukast group based on the biochemical examination and there was no significant difference between NAC and Sham groups. Mean antioxidant SH amount was detected as 50.94±8.58 in NAC group and there was no eloquent difference from montelukast and sham groups (p> 0.05). But there was an eloquent difference in the mean antioxidant SH quantity of placebo and montelukast groups and NAC and sham groups (p <0.05).

Mean MDA amounts demonstrating oxidative substance were detected as 4.46±0.25 in montelukast group and as 4.04±0.43 in NAC group. MDA amount was detected significantly lower than the Placebo group in Montelukast and NAC groups (p <0.05). There was no eloquent difference in MDA amounts among Montelukast, NAC and Sham groups (p> 0.05).

In biomechanical terms, mean amounts of MPO, which is another oxidative substance, were detected as 0.073±0.004 in montelukast group and as 0.062±0.011 in NAC group. MPO amount was detected significantly lower in Montelukast and NAC groups than the Placebo group (p <0.05). There was no eloquent difference when Montelukast and NAC groups were compared for MPO amounts (p> 0.05).

Total nitrite amounts demonstrating free oxygen radical formation in the tissue were detected as 0.050±0.005 in montelukast group and as 0.049±0.010 in the NAC group. An eloquent difference was detected among Montelukast and NAC groups and the placebo group (p <0.05). Total nitrite quantities of Montelukast, NAC and Sham groups were not eloquent different (p> 0.05).

When the blood urea levels of the groups were compared, placebo and sham groups were statistically eloquent different (p<0.05) and there was no statistically meaningful difference compared to the NAC group. There was a statistically eloquent difference between the NAC group and the placebo group (p<0.05), while there was no statistically meaningful difference among other groups (p>0.05).

Mean creatinine levels were eloquent different among the montelukast and placebo groups (p<0.05). Other groups were not meaningfully different (p>0.05). There was an eloquent difference between NAC group and placebo group (p<0.05) while there was no eloquent difference between the others (Table 2).

**Table 2:** Group comparisons by biochemical assessment.

	SH (nmol/mg protein) (Mean[SD])	MDA (µmol/g wet tissue) (Mean[SD])	MPO (Δabs/min/ g tissue) (Mean[SD])	Total Nitrite (nmol/mg wet tissue) (Mean[SD])	Urea mg/dL (Mean[SD])	Kreatin mg/dL (Mean[SD])
Montelukast	(45.09[6.73])	(4.46[.25])	(.073[.004])	(.050[.005])	(103.33[5.27])	(1.02[.064])
NAC	(50.94[8.58])	(4.04[.43])	(.062[.011])	(.049[.010])	(113.33[13.54])	(1.10[.104])
Placebo	(97.09[19.70])	(6.92[.99])	(.134[.030])	(.070[.006])	(180.0[11.05])	(1.48[.058])
Sham treated	(39.22[4.31])	(5.70[1.13])	(.0460[.003])	(.032[.006])	(45.0[2.88])	(.766[.033])
*P Value*:*						
Montelukast vs placebo	.002	.027	.024	.038	.000	.000
Montelukast vs NAC	.536	.145	.354	.565	.266	.562
NAC vs placebo	.005	.003	.015	.031	.003	.012
Montelukast vs sham treated	.782	.229	.033	.052	.012	.060
NAC vs sham treated	.309	.309	.354	.354	.051	.112

*For SH, MDA, MPO and total nitrite overall group comparisons Mann-Whitney U and Kruskal wallis test *P<.05.*

### DMSA renal scan

All rats had DMSA renal scintigraphy pre and post-surgically. There was no eloquent difference in pre-surgery scintigraphy results among the groups. In the montelukast group, the DMSA renal scintigraphy measurements made after 6h reperfusion, the renal function levels were significant as per the placebo and sham groups (p<0.05) and there was no difference as per the NAC group (p>0.05).

The DMSA worth in the NAC group was eloquently different from placebo and sham groups (p<0.05) (Table 1).

## Discussion

Current data show that in conditions of temporary renal ischaemia, structural and functional kidney damage is formed associated with an increase in pro-inflammatory cytokines. The CysLT_1_ receptor antagonist, montelukast sodium, reduces the concentrations of these pro-inflammatory cytokines, reducing the severity of the damage by increasing the anti-oxidative capacity^6^.

The cause of renal I/R damage is usually multifactorial as a mechanism including mutually dependent inflammatory response, free radical damage and hypoxia^7^. Cell damage induced by oxygen deprivation has been shown in the pathogenesis together with a series of processes^8^.

These processes include reduced cell volume, irregularity in cell calcium metabolism, activation of phospholipases resulting in the manufacturing of harmful lipid metabolites, and the production of free radicals. We can expect free-oxygen-radical-mediated cell injury when oxygen support is provided to the tissue with reperfusion and when radical oxygen creation overrun the renal capacity, which has high cellular detoxification capability. Determining the amount of oxygen radicals is difficult because of their short life and reactive nature^9,10^. With an increase in toxic oxygen metabolites, while a significant increase is seen in renal MDA levels showing increased lipid peroxidization associated with I/R damage, the tissue glutathione levels are decreased showing the antioxidant pool has been used up. In several studies, a relationship was shown between Ischemia/Reperfusion damage in the kidney and lipid peroxidization, which is an autocatalytic contraption causing oxidative ravage in the cell membranes^11-13^.

Montelukast reduces oxidative damage in the cellular structure. The amount of glutathione, which is an intracellular antioxidant, does not change. Therefore, it is thought that the antioxidant effect of montelukast is formed with peroxidation without consuming the GSH stock in the tissue, and this is supported with montelukast activation in the antioxidant pool. In addition, the effect of I/R inducing a total antioxidant capacity reduction is reversed with montelukast treatment. Furthermore, as the effect of the level of montelukast in the tissue causes a dramatic reduction in serum LDH activity, LDH is a marker of prevalent tissue damage. Consistent with these biochemical alterations, morphological assessment of the tissues has revealed the protective effect of montelukast against degenerative changes in the kidney caused by I/R. The protective effect of montelukast against oxidative damage occurs together with healing in renal dysfunction^6^.

Neutrophils are considered to show a strengthening effect in the initial reperfusion reaction. It must also be taken into consideration that neutrophils play an important role since they are responsible for the injury cascade loaded on top of reperfusion. It has been shown that I/R causes an acute inflammatory response characterized by neutrophil activation^14^.

An increase in MPO activation levels is a tissue neutrophil infiltration index. In a previous study, low MPO levels and infiltration were inhibited with montelukast treatment and montelukast’s protective effect was shown to be neutrophil-dependent^6^. Ischaemia is known to increase cytosolic calcium concentration, and this leads to increased lipoxygenase activity, an increase in phospholipase A_2_ and production of leukotrienes. As CysLTs can increase chemotactic and chemokinetic properties and vascular permeability, they also activate inflammatory mediators^3^. In a study by Takamatsu *et al.* ^15^ on the act of leukotrienes in hepatic I/R, it was shown that these lipid mediators increase vascular permeability, which is a common feature of I/R damage and that they also increase neutrophil supplementation. In the same way, it has been represented that the significant amount of CysLTs produced in the brain during cerebral ischaemia disrupted the blood brain barrier and caused tissue oedema^16^.

In contrast, it has also been shown that prontolukast and montelukast in cerebral ischaemic rats and mice decreased the infarct volume and reduced neurological deficits^17,18^. In a study by Patel *et al*.^19^ it was observed that less renal damage developed in 5-lipoxygenase-suppressed experimental mice than wild-type mice. It was also shown that the damage caused by ischaemia reperfusion recovered with zileuton which is a 5-lipoxygenase inhibitor. In a hepatic I/R model, Matsui *et al*.^20^ demonstrated that while ardisiaquinone, a 5-lipoxygenase inhibitor, reduced tissue MPO activity, there was also an improvement in hepatic functions. In another study, the CysLT receptor antagonist, CP 105,696, was found to be efficient versus local, distant and systemic inflammatory changes in I/R-mediated violent intestinal damage^21^. Noiri *et al*.^22^ showed that an important protective effect was provided by CysLTs receptor antagonist in acute renal failure. Together with all these previous studies, as shown in the study by Şener *et al*.^6^, it was seen in the current study that I/R-mediated oxidative damage and the correlated organ dysfunction can be corrected with CysLTs receptor blockage, as CysLTs are responsible for macrophage supplementation and neutrophils that produce proinflammatory mediators and increase permeability. In an animal model of human membranous nephropathy, it was shown that LTD4 was synthesized from macrophages or taken with direct origin from macrophage-originated LTA4, while LTA4 was synthesized from glomerular cells^23^. In this case, it can be said that the synthesis of CysLTs is increased from macrophages in an ischaemic state, in fact that they are synthesized from ischaemic renal tissue and this is related not to supplementation of macrophages and neutrophils by the CysLT receptor antagonist, montelukast, but to the receptors also found in mesengial cells and/or macrophages^6^.

In a study by Pompermayer *et al*.^24^, it was shown that neutrophil accumulation and cytokine content, TNF-a, IL-1 and IL-10, rised in reperfused kidneys. Similarly, Teng *et al*.^25^ evaluated the act of cytokines in I/R damage in lung tissue and showed that I/R caused the emergence of TNF-a, IL-1, IL-6, IL-10 and IFN-gamma in an isolated perfusion rat lung model and the emerging cytokines could exacerbate I/R damage. Correlated to those results, Kher *et al*.^26^ also showed that TNF-a, IL-1 and IL-6 increased after renal I/R. In a study by Şener *et al*.^6^, it was shown that montelukast decreased inflammatory parameters such as TNF-a, IL-1, IL-6 and LTB_4_ in the protection of I/R-mediated renal damage.

Anderson *et al*.^27^ showed that the proinflammatory activity of neutrophils was inhibited with the c-AMP-dependent mechanism of montelukast. Mederios *et al*.^28^ examined the renal protective effect of sildenafil with scintigraphic imaging and reported that it was protective against I/R damage. In a study by Fidillioğlu *et al*.^29^, melatonin’s protective effect against I/R damage was investigated and it was shown to be protective towards I/R damage in the kidneys and the liver.

Gideroğlu *et al*.^30^ demonstrated that skin flap survival in rats was prolonged by montelukast reversing the I/R oxidation response, inhibiting neutrophil infiltration and providing oxidant-antioxidant balance. In another study by Şener *et al*.^31^, montelukast was shown to have a protective effect towards oxidant harm and bladder dysfunction caused by I/R. Erdoğan *et al*.^32^ demonstrated that erdostine and NAC were protective against renal I/R damage. Sirmali *et al*.^33^ examined the protective effect of Vitamin C, Vitamin E and erdostine against renal I/R damage and showed that a nephroprotective effect was provided by reducing oxidative stress.

In the light of all this information, and as a result of the tissue, biochemical and scintigraphic tests applied in the current study, montelukast showed a protective impact towards renal I/R damage, which is often encountered in clinical urology practice. The results of the current study are consistent with the findings of the above-mentioned studies.

## Conclusions

The tissue level with biochemical data and imaging methods clearly showed that montelukast (CysLT1 receptor antagonist) reduced the neutrophil accumulation, oxidative damage and renal dysfunction that occurred following renal I/R, and as a result it can be concluded that CysLTs are tissue damage mediators following renal I/R.

According to the current study’s findings, the application of NAC and Montelukast Sodium following the development of renal injury associated with long warm renal ischaemia has a nephroprotective effect. The effect expected at the beginning was accomplished but further studies are still needed on this subject.
